# Supplementary material for: Enough to Feed Ourselves!—Food Plants in Bulgarian Rural Home Gardens
Source: Plants (Basel). 2021 Nov 19;10(11):2520. doi: 10.3390/plants10112520 (PMC8623271; doi:10.3390/plants10112520)
Supplement: Supplementary file 1 [file plants-10-02520-s001.zip › plants-1456675-supplementary.pdf]

Table S1. Food, medicinal and fodder plants cultivated in Bulgarian rural home gardens.

Plant taxon	Family	Vernacular Name	Part Used	Consumption	Preservation[1]	Provinces[2]	Occurrence[3]
Abelmoschus esculentus (L.) Moench	Malvaceae	bamya, bamnya	fruits	stews and pickles	D, F, FR	Hs, Pd	8
**Actinidia deliciosa (A.Chev.) C.F.Liang & A.R.Ferguson	Actinidiaceae	kivi	fruits	fresh, spirits		Bl, Hs, Mn, Sm	11
Agastache foeniculum (Pursh) Kuntze	Lamiaceae	lophant	herbage	herbal tea	D	Hs	2
Allium ampeloprasum L.	Amaryllidaceae	praz	pseudostem, leaves	raw or cooked, pastries, flavoring for cured sausages		Bl, Hs, Mn	15
Allium cepa L. Aggregatum Group	Amaryllidaceae	bogat luk	bulb, leaves	raw or cooked		Lv, Mn, Pd	3
Allium cepa L.							
Cepa Group	Amaryllidaceae	luk, arpadzhikov luk, kromid, sugan	bulb, leaves	raw or cooked, pastries, preserves	P	All	65
Allium cepa L. Proliferum Group	Amaryllidaceae	jivorazhdasht luk	bulb, leaves	raw or cooked		Mn, Pd, Vr	3
Allium sativum L.	Amaryllidaceae	chesan, byal luk	bulb	raw or cooked, preserves	P	All	65
Allium schoenoprasum L.	Amaryllidaceae	rezanets	pseudostem, leaves	salads		Sm	1
Allium tuberosum Rottler ex Spreng.	Amaryllidaceae	chesnova treva	pseudostem, leaves	raw or cooked		Mn	3
Allium ursinum L.	Amaryllidaceae	levurda, div chesan	leaves	raw or cooked, pickles, alkohol extracts		Mn	2
Allium vineale L.	Amaryllidaceae	div luk	leaves	salads		Sm	1
Anethum graveolens L.	Apiaceae	kopar	leaves, inflorescences, seeds	flavoring of various dishes and preserves	D, P, F, FR	Bl, Hs, Lv, Mn, Pd, Sm, Vr	27
*Anthriscus cerefolium (L.) Hoffm.	Apiaceae		herbage	herbal tea		Sm	1
Apium graveolens L.	Apiaceae	tselina, kereviz, kirviz	herbage, taproot	raw or cooked, preserves	D, F, FR	Bl, Hs, Lv, Pd, Sm, Vr	17
Arachis hypogaea L.	Fabaceae	fustuk	seeds	rosted, snack, dessert	D	Pd, Bl	2
Armoracia rusticana P.Gaertn., B.Mey. & Scherb.	Brassicaceae	hryan	rhizomes	preserves	F, FR	Bl, Hs, Lv, Mn, Pd	16
Aronia melanocarpa (Michx.) Elliott	Rosaceae	aronia	fruits	jam, compote, beverages	P, F	Bl, Hs, Lv, Mn, Pd, Sm	19
*Artemisia absinthium L.	Asteraceae	pelin	herbage	flavoring for wines		Bl, Hs	2
Artemisia pontica L.	Asteraceae	pelin, kadunska kalka, piy pak	herbage	flavoring for wines and		Bl, Hs, Lv,	9

				spirits		Mn, Pd	
Asparagus officinalis L.	Asparagaceae	asperzha	young shoots	cooked		Bl, Mn	
Atriplex hortensis L.	Chenopodiacea	loboda	leaves	cooked		BL, Lv, Sm	
Beta vulgaris L. ssp. vulgaris							
Altissima Group	Amaranthaceae	zaharno tsveklo	taproot	preserves (madzhun, petmez)	P	Bl, Hs, Bd, Sm	7
Beta vulgaris L.							
ssp. vulgaris							
Conditiva Group	Amaranthaceae	cherveno tsveklo, chekane, kochan	leaves, taproot	raw and cooked, pastries, pickles	F	Bl, Hs, Lv, Mn, Pd, Sm	19
Beta vulgaris L. ssp. vulgaris Cicla Group	Amaranthaceae	zimen spanak, mangold	leaves	cooked	P	Hs, Lv, Mn, Sm	6
Beta vulgaris L. ssp. vulgaris Vulgaris Group	Amaranthaceae	furazhno tsveklo	leaves, taproot	cooked, pastries, fodder		Bl, Hs, Sm	5
Brassica napus L. Napobrassica Group	Brassicaceae	kotchan, kotchen	leaves, stem	flavoring for bean stews		Sm	1
Brassica oleracea L.							
var. italica Plenc	Brassicaceae	broccoli	inflorescence	raw or cooked	FR	Bl, Sm	2
Brassica oleracea var. capitata L.	Brassicaceae	glavesto zele	leaves	raw and cooked, pickles	P, F	Bl, Hs, Lv, Pd, Sm	19
Brassica oleracea var. gongylodes L.	Brassicaceae	alabash, kotchan, kholrabi	stem	raw or cooked, pickles		Bl, Pd, Sm	8
Calendula officinalis L.	Asteraceae	neven	flowers	herbal tea, oil extract	D	Hs, Lv, Mn, Sm	5
*Capsella bursa-pastoris (L.) Medik.	Brassicaceae	ovcharska torbichka, nekutche	herbage	pastries	FR	Hs	1
Capsicum annuum L. Cultivars	Solanaceae	chushka, piper, puper, kapitsi	fruits	raw, cooked or roasted, pickles, paprika powder	D, P, F, FR	All	65
Capsicum baccatum var. pendulum L.	Solanaceae	kambanki	fruits	flavoring for various dishes	D	Hs	1
Castanea sativa Mill.	Fagaceae	kesten	seeds	cooked, snacks, preserved	D, P	Bl	1
Cicer arietinum L.	Fabaceae	nakhut, lakhut	seeds	cooked or roasted (snack)	D	Hs	2
Citrullus lanatus (Thunb.) Matsum. & Nakai	Cucurbitaceae	dinya, karpuza, lyubenitsa	fruits	fresh, preserves	P	Bl, Hs, Pd	10
**Citrus limon (L.) Osbeck	Rutaceae	limon	fruits	fresh, flavoring for preserves	P	Bl, Hs, Lv, Pd, Sm	17

Coriandrum sativum L.	Apiaceae	gudecha merudiya	seeds	flavoring for cured sausages	D	Bl	1
Cornus mas L.	Cornaceae	dryan	fruits	row, preserves, spirits	D, P	Hs, Lv, Pd, Sm	15
Corylus avellana L.	Betulaceae	leska	seeds	snack, desserts	D	Bl, Hs, Lv, Mn, Sm	9
Corylus colurna L.	Betulaceae	turska leska	seeds	snack, desserts	D	Mn	1
Corylus maxima Mill.	Betulaceae	tsarigradska leska	seeds	snack, desserts	D	Hs	1
Cucumis melo L.	Cucurbitaceae	pupesh, kaun, pipon	fruits	fresh, preserves, spirits	P	Hs	7
Cucumis sativus L. Cultivars	Cucurbitaceae	krastavitsa	fruits	fresh, preserves, pickles	P, F	All	65
Cucurbita maxima Duchesne	Cucurbitaceae	byala tikva	fruits, flowers, stem tops	raw and cooked, pastries, desserts, preserves, snack (seeds)	P	Bl, Hs, Mn, Sm	12
Cucurbita moschata Duchesne	Cucurbitaceae	tikva tsigulka	fruits, flowers, stem tops	raw and cooked, pastries, desserts, preserves, snack (seeds)	P	Bl, Hs, Sm	15
Cucurbita pepo L.	Cucurbitaceae	tikvichka, balka	fruits, flowers, stem tops	raw and cooked, preserves, spirits	P	All	64
Cucurbita pepo L. var. styriaca	Cucurbitaceae	tikva, svinska tikva	fruits, flowers, stem tops	raw and cooked, pastries, desserts, preserves, snack (seeds)	P	Bl, Hs, Lv, Pd, Sm	10
Cydonia oblonga Mill.	Rosaceae	dyulya, dunkya	fruits	fresh, preserves, flavoring for pickles	P, F	Bl, Hs, Lv, Mn, Pd, Sm,Vr	23
Daucus carota L.	Apiaceae	morkov	taproot	raw and cooked, preserves, pickles	P, F	Bl, Hs, Lv, Mn, Pd, Sm,Vr	24
**Diospyros kaki L.f.	Ebenaceae	rayska yabalka	fruits	raw, preserves	P	Bl, Hs, Pd	5
Elaeagnus angustifolia L.	Elaeagnaceae	brashlyanka, brashnyanka	fruits	raw (snack)		Hs	2
**Eriobotrya japonica (Thunb.) Lindl.	Rosaceae	yaponska mushmula	fruits	raw, preserves	P	Bl, Hs, Mn	7
Eruca vesicaria (L.) Cav.	Brassicaceae	rukola	leaves	raw		Lv, Mn	3
**Ficus carica L.	Moraceae	smokinya, smokva	fruits	fresh, preserves, spirits	D, P	All	27
Foeniculum vulgare Mill.	Apiaceae	div kopar, rezene	leaves, inflorescenses, seeds	raw, pickles, flavoring for cured sausages	D, P, F	Lv, Mn, Pd	6
*Fragaria vesca L.	Rosaceae	diva yagoda	fruits	fresh, preserves, herbal	D, P	Lv, Mn, Sm	4

				tea			
Fragaria x ananassa (Duchesne ex Weston) Duchesne ex Rozier	Rosaceae	yagoda	fruit	fresh, preserves	P, FR	Bl, Hs, Lv, Mn, Pd, Sm,Vr	37
Galanthus elwesii Hook.f.	Amaryllidaceae	kokitche	leaves, flowers	alcoholic extract		Hs	1
Geranium macrorrhizum L.	Geraniaceae	zdravets	leaves	fresh		Hs, Mn, Sm	3
Helianthus annuus L.	Asteraceae	slanchogled	seeds	snack (roasted)	D	Hs	2
Helianthus tuberosus L.	Asteraceae	zemna yabalka, guliya	tubers	raw, pickles	F	Bl, Hs, Lv, Sm, Vr	7
Juglans regia L.	Juglandaceae	orekh	seeds, young fruits	snack, desserts, preserves and alcohol extract (young fruits)	D, P	Bl, Hs, Lv, Mn, Sm,Vr	14
Lactuca sativa var. crispa (L.) Schübl. & G.Martens Cultivars	Asteraceae	salata, kadrava marulya	leaves	raw (salads)		All	40
Lactuca sativa var. longifolia (Lam.) Alef. Cultivars	Asteraceae	marulya, byala marulya	leaves	raw (salads), pastries		All	45
Lathyrus sativus L.	Fabaceae	star grakh	seeds	cooked	D	Hs	1
**Laurus nobilis L.	Lauraceae	dafina	leaves	flavoring for meat and vegetable dishes	D	Bl, Hs, Lv	9
Lavandula angustifolia Mill.	Lamiaceae	lavandula	herbage	herbal tea	D	Hs, Lv	2
Lens culinaris Medik.	Fabaceae	leshta	seeds	cooked	D	Hs	2
Levisticum officinale W.D.J.Koch	Apiaceae	devesil, lyushtyan	leaves	flavoring of various dishes	D, FR	Hs, Lv, Mn, Pv, Sm,Vr	16
Lilium candidum L.	Liliaceae	byal krem, zambak	flowers	oil extract		Hs	1
Lycium barbarum L.	Solanaceae	merdzhan, gojiberry	fruits	raw	D, FR	Bl, Hs, Lv, Mn, Pd, Sm	11
Lycopersicon esculentum Mill. Cultivars	Solanaceae	domat	fruits	raw or cooked, preserves, pickles	D, P, F	All	65
Malus domestica Borkh.	Rosaceae	yabalka	fruits	raw or cooked, preserves, vinegar, pastries, spirits	D, P, F	All	48
Medicago sativa L.	Fabaceae	lyutserna	herbage	fodder	D	Hs, Sm	4
Melissa officinalis L.	Lamiaceae	matochina	herbage	herbal tea, beverages, syrup	D, P	Bl, Hs, Lv, Mn, Pd, Sm	17
Mentha spicata L.	Lamiaceae	gyozum, dzhodzhen	herbage	flavoring for various	D, FR	All	47

				dishes, pastries			
Mentha x piperita L.	Lamiaceae	mentha	herbage	herbal tea, beverages, syrup	D	Bl, Hs, Lv, Mn, Pd, Sm	15
Mespilus germanica L.	Rosaceae	mushmula	fruits, timber	raw, preserves, flavoring and coloring of spirits (timber)	P	Bl, Hs	2
Morus alba L.	Moraceae	chernitsa, byala duda	fruits	raw, preserves	P	Hs, Lv	5
**Morus nigra L.	Moraceae	chernitsa, cherna duda	fruits	raw, preserves	P	Hs	1
Nectaroscordum siculum subsp. bulgaricum (Janka) Stearn	Amaryllidaceae	samardala	leaves	herbal salt	D	Mn	1
Ocimum basilicum L.	Lamiaceae	bosilek	herbage	herbal tea, flavoring for salads and pizza	D	Bl, Hs, Lv, Pd, Sm,Vr	19
Ocimum minimum L.	Lamiaceae	grutski bosilek	herbage	herbal tea, flavoring for salads and pizza		Bl, Hs, Sm	4
**Origanum vulgare subsp. hirtum (Link) Ietsw.	Lamiaceae	byal rigan, visoka rigan	herbage	flavoring for various dishes, meat products and preserves	D	Hs, Lv, Sm	12
*Papaver rhoeas L.	Papaveraceae	kadunka, bulitsa, mak, machek	herbage	pastries	FR	Hs	2
Papaver somniferum L.	Papaveraceae	mak	seeds	pastries	D	Hs, Lv, Mn, Sm, Vr	6
Pastinaca sativa L.	Apiaceae	pashtarnak	taproot	flavoring for various dishes	FR	Hs, Lv, Pd, Sm	6
Pelargonium roseum Willd.	Geraniaceae	indrishe	leaves	flavoring for fruit preserves, herbal tea		Bl, Hs, Lv, Pd	10
Petroselinum crispum (Mill.) Fuss	Apiaceae	magdanoz, merudiya	herbage	flavoring for various dishes and pickles	D, FR	All	65
Phaseolus coccineus L.	Fabaceae	fasulevitsa, bivolar, Smilyanski fasul	seeds	cooked	D	Bl, Hs, Lv, Sm	14
Phaseolus lunatus L.	Fabaceae	popski fasul	seeds	cooked	D	Pd	1
Phaseolus vulgaris L.	Fabaceae	fasul, bob, kapichki	seeds, pods	cooked	D	All	44
Physalis peruviana L.	Solanaceae	grutska smokinya	fruits	fresh		Bl	1
*Phytolacca americana L.	Phytolaccaceae	butima, pishalo	leaves	cooked (sarmi)	D	Bl	1
Pisum sativum L.	Fabaceae	grakh	seeds	cooked, preserves	P, FR	Hs, Lv	2
*Portulaca oleracea L.	Portulacaceae	tutchenitsa, semzelek	herbage	raw or cooked, pastries		Bl, Hs	4
Prunus armeniaca L.	Rosaceae	kaysiya	fruits	fresh, desserts,	D, P	Bl, Hs, Lv,	16

				preserves, spirits		Mn, Pd	
Prunus avium (L.) L.	Rosaceae	cheresha	fruits, leaves	fresh, desserts, preserves, spirits	P	All	31
Prunus cerasifera Ehrh.	Rosaceae	dzhanka, sliva	fruits	fresh, preserves, pickles, spirits	P, F	Bl, Hs, Lv, Sm, Vr	9
Prunus cerasus L.	Rosaceae	vishnya	fruits, leaves	fresh, desserts, preserves, liquer	P	Bl, Hs, Lv, Mn, Sm	11
Prunus cerasus L. x P. avium (L.) L.	Rosaceae	vishnap	fruits	fresh, desserts, preserves	P	Hs	1
Prunus domestica L.	Rosaceae	sliva, sinya sliva, chernoslivka	fruits	fresh, desserts, preserves, spirits	D, P	Bl, Hs, Lv, Pd, Sm	24
**Prunus dulcis (Mill.) D.A.Webb	Rosaceae	badem	seeds	fresh, desserts	D	Hs, Sm	7
Prunus persica (L.) Batsch	Rosaceae	praskova	fruits	fresh, desserts, preserves	P	Bl, Hs, Lv, Mn, Pd, Sm, Vr	35
Prunus persica (L.) Batsch var. nectarina (Aiton) Maxim	Rosaceae	nectarina	fruits	fresh, desserts, preserves	P	Sm	1
**Punica granatum L.	Rosaceae	nar, kalinka	fruits	fresh, desserts	P	Bl, Hs, Pd	6
Pyrus communis L.	Rosaceae	krusha	fruits	fresh, desserts, preserves, spirits	D, P	Bl, Hs, Lv, Mn, Pd, Sm,Vr	37
*Ranunculus arvensis L.	Ranunculaceae	pateshko krache	herbage	pastries		Hs	1
Raphanus raphanistrum subsp. sativus (L.) Domin	Brassicaceae	repichka	taproot	raw (salads)		All	64
Ribes aureum Pursh	Grossulariaceae	zhulto frensko grozde, zlaten kasis	fruits	fresh, desserts, preserves	P, FR	Hs, Pd	6
Ribes nigrum L.	Grossulariaceae	kasis	fruits	fresh, desserts, preserves, spirits	P, FR	Bl, Lv, Mn, Sm	4
Ribes rubrum L.	Grossulariaceae	frensko grozde	fruits	fresh, desserts, preserves	P, FR	Bl, Hs, Lv, Mn, Pd, Sm	17
Ribes uva-crispa L.	Grossulariaceae	tsarigradsko grozde	fruits	fresh, desserts, preserves	P, FR	Hs, Lv, Mn	3
Rosa sp. Cultivars	Rosaceae	rosa, gyul, trendafil	flowers	herbal tea, preserves	D, P	Hs, Lv	2
**Rosmarinus officinalis L.	Lamiaceae	rozmarin	leaves	flavoring for meat	D	Hs	2
Rubus caesius L.	Rosaceae	kapina	fruits	fresh, desserts, preserves	P, FR	Bl, Hs, Lv, Mn, Pd, Sm, Vr	13

Rubus idaeus L.	Rosaceae	malina	fruits	fresh, desserts, preserves	P, FR	All	31
Rubus illecebrosus Focke	Rosaceae	yagodomalina	fruits	fresh, desserts	P, FR	Lv	1
Rumex acetosa L.	Polygonaceae	kiselets, kiselek	leaves	cooked, pastries	D	Hs, Lv, Mn, Sm	8
*Rumex patientia L.	Polygonaceae	lapad	leaves	cooked, pastries		Hs, Mn	4
Salvia officinalis L.	Lamiaceae	gradinski chai	leaves	herbal tea	D	Bl, Hs, Lv, Sm	11
*Sambucus nigra L.	Adoxaceae	baz, buz	flowers, fruits	herbal tea, syrup, preserves, beverages	D, P, F	Hs	1
Satureja hortensis L.	Lamiaceae	chubritsa, chubrika	herbage	flavoring for various dishes and cured sausages	D	All	28
Sideritis scardica Gris.	Lamiaceae	pirinski chai	herbage	herbal tea	D	Hs, Sm	4
*Silybum marianum (L.) Gaertn.	Asteraceae	byal trun	fruits	herbal tea		Mn	1
Solanum melongena L.	Solanaceae	patladzhan, sin domat	fruits	cooked or roasted, preserves	P	Bl, Hs, Pd, Pv	13
Solanum tuberosum L.	Solanaceae	kartof, kompir, baraboshki	tubers	cooked or roasted		All	
Spinacia oleracea L.	Amaranthaceae	spanak	leaves	raw or cooked	FR	Hs, Lv, Sm	13
*Stellaria media (L.) Vill.	Caryophyllaceae	zhabni chrevtsa	herbage	pastries	FR	Hs	1
Tagetes erecta L.	Asteraceae	turta, fundi	flowers	herbal tea	D	Bl, Hs	2
Tanacetum balsamita L.	Asteraceae	kaloferche, kalofer	leaves	flavoring for various dishes		Lv	1
Thymus sp.	Lamiaceae	mashterka bilka	herbage	herbal tea	D	Hs, Mn, Sm	
Thymus vulgaris L.	Lamiaceae	mashterka podpravka	herbage	flavoring for various dishes	D	Pd, Sm	2
Tilia tomentosa Moench	Tiliaceae	lipa	flowers	herbal tea	D	Hs, Lv, Mn, Sm	5
Trigonella caerulea (L.) Ser.	Fabaceae	smindukh, smindol, kvachka	herbage, seeds	flavoring for various dishes, herbal salt	D	Hs, Lv, Pd	3
*Urtica dioica L.	Urticaceae	kopriva	herbage	cooked	D	Hs, Mn	3
Vaccinium corymbosum L.	Ericaceae	amerikanska borovinka	fruits	fresh	Fr	Mn	1
Valeriana officinalis L.	Caprifoliaceae	valeriana	rhizomes	herbal tea, alcohol extract	D	Lv	1

* - Semi-cultivated species'; **Mediterranean or subtropical taxa. [1]Preservation modes: D – drying; P – preserve (sterilized, marinated or boiled); F – fermented; FR – frozen. [2] Province: Bl – Blagoevgrad; Hs – Haskovo; Lv – Lovech; Mn – Montana; Pv – Pleven; Pd – Plovdiv; Sm – Smolyan; Vr – Vratsa. [3] Occurrence – Number of gardens (N = 65).

Table S2. Preference to crop groups as parts of the food growing area (FGA) across studied home gardens.

Crop group	0 FGA[1]		< 1/5 FGA		1/5 - 1/3 FGA		> 1/3 FGA	
	LL[2]	SMM[3]	LL	SMM	LL	SMM	LL	SMM
Fruiting vegetables*	0	0	1	5	26	19	5	9
Bulbous vegetables[NS]	0	0	17	18	13	12	2	2
Herbs and Spices[NS]	0	0	30	33	1	0	0	0
Leaf, stalk and stem vegetables[NS]	0	1	30	31	1	1	0	0
Root & Tuber vegetables[NS]	3	1	21	25	5	7	0	0
Legumes[NS]	4	5	25	20	2	6	0	0
Berries & Small fruits[NS]	4	4	26	24	2	4	0	0
Cereals (Corn) [NS]	19a	16	9	13	0	3	0	0
Animal fodder[NS]	28	29	2	1	2	3	0	0

[1] Parts of food growing area (FGA) occupied by each crop group; [2] Lowland settlements (LL), n = 31; [3]Semi-mountainous and mountainous settlements (SMM), n = 33; *Fisher's exact tests *p*-values ≤ 0.05, NS – not significant.
